# Pexidartinib synergize PD-1 antibody through inhibiting treg infiltration by reducing TAM-derived CCL22 in lung adenocarcinoma

**DOI:** 10.3389/fphar.2023.1092767

**Published:** 2023-03-08

**Authors:** Wei Zhang, Xi Jiang, Youcheng Zou, Lihua Yuan, Xiaobo Wang

**Affiliations:** ^1^ Emergency and Disaster Medical Center, The Seventh Affiliated Hospital, Sun Yat-Sen University, Shenzhen, China; ^2^ Clinical Laboratory, The Seventh Affiliated Hospital, Sun Yat-Sen University, Shenzhen, China; ^3^ Emergency Department, Shenzhen Longgang Central Hospital, Shenzhen, China; ^4^ Department of Pediatric Surgery, University of Hong Kong-Shenzhen Hospital, Shenzhen, China; ^5^ Department of Hematology, The Seventh Affiliated Hospital, Sun Yat-Sen University, Shenzhen, China

**Keywords:** pexidartinib, PD-1, treg, CCL22, tumor associated macrophage, lung adenocarcinoma

## Abstract

There is a crosstalk between Tumor-associated macrophages (TAM) and tumor-infiltrating T cells in tumor environment. TAM could inhibit the activity of cytotoxic T cells; TAM could also regulate the composition of T cells in tumor immune environment. The combination therapy for TAM and tumor infiltrated T cells has been widely noticed, but the crosstalk between TAM and tumor infiltrated T cells remains unclear in the process of combination therapy. We treated lung adenocarcinoma tumor models with pexidartinib, which targets macrophage colony stimulating factor receptor (M-CSFR) and c-kit tyrosine kinase, to inhibited TAM. Pexidartinib inhibited the ratio of macrophages in the tumor and also altered macrophage polarization. In addition to reprogram TAM, pexidartinib also changed the composition of tumor-invasive T cells. After pexidartinib treatment, the total number of T cells, CD8^+^ T cells and Treg cells were all decreased, the ratio of CD8+T/Treg increased significantly. According to the analysis of cytokines and chemokines during the treatment of pexidartinib, CCL22, as a chemokine for Treg recruitment, significantly decreased after the treatment of pexidartinib. Base on the above observation, the combination of pexidartinib and PD-1 antibody were used in the treatment of lung adenocarcinoma subcutaneous tumor model, the combination therapy has significantly improved the efficacy of tumor treatment compared with the monotherapy. Meanwhile, compared with pexidartinib monotherapy, the combination treatment further switches the polarization status of tumor-associated macrophages. In summary, our results showed that the combination of pexidartinib and PD-1 antibody showed a synergy and significantly improved the anti-tumor efficacy, through pexidartinib increasing CD8T/Treg ratio by reducing TAM-derived CCL22.

## Introduction

Globally, lung cancer accounted for about 11.4% of all new tumors in 2020, and accounted for 18% of all cancer deaths, making it the leading cancer cause of death (Sung et al., 2021). Non-small cell lung cancer (NSCLC) comprise 85% of all lung cancers (Jemal et al., 2011), and the 5 years survival rate is 25% (Viale, 2020). Researchers have established a variety of approaches for the treatment of NSCLC, such as chemotherapy, targeted therapy and immunotherapy.

Tumor immunotherapy is one of the most advanced tumors therapies. A large number of immunotherapy methods such as PD-1, CTLA-4 have been applied to NSCLC which have produced obvious positive outcome (Antonia et al., 2016; Garon et al., 2019). Among them, the anti-tumor effect of PD-1 antibody which could activate immune suppressed T cells has been widely verified in a variety of solid tumors (Robert et al., 2015; Herbst et al., 2016), also in NSCLC (Reck et al., 2016; Horn et al., 2017). Notably, PD-1/PD-L1 has only 20 to 30 percent response rate when it is used as a monotherapy (Topalian et al., 2015). In addition to T cells, there are a large number of immune cells in the tumor immune microenvironment, including tumor-associated macrophage (TAM), myeloid-derived suppressor cells (MDSC), B cells, dendritic cells (DC) *etc.*, which play different roles in the process of PD-1 antibody treatment. TAM is a kind of immune cell with a large proportion in solid tumors, which usually plays a role in promoting tumor development in solid tumors and leads to worse prognosis (Gentles et al., 2015). What’s more, the crosstalk between TAM and T cells usually leads to the dysfunction of T cells. Therefore, T cell-involved immunotherapy combined with TAM-target immunotherapy is a very effective and promising therapy strategy (Wynn et al., 2013; Chen et al., 2021).

Colony-stimulating factor 1 receptor (CSF1R,CSF1-R, CSF-1R) is a high expression receptor on the surface of TAM, which can promote TAM migration, differentiation and survival (Hume and MacDonald, 2012). CSF1R belongs to the platelet-derived growth factor receptor family, which is a type III receptor tyrosine kinase (Cannarile et al., 2017). CSF1 and IL-34 are two ligands of CSF1R. Neutralization of CSF1R or CSF1 has been shown to inhibit tumor growth in a variety of tumor types by inhibiting the proliferation of intratumor macrophages (Strachan et al., 2013; Xu et al., 2013).

PLX3397 (pexidartinib) is an inhibitor which targets CSF1R and c-kit tyrosine kinase. PLX3397 is safe and effective in reducing TAMs in several solid tumor types (Tap et al., 2015; Butowski et al., 2016). It has been reported that PLX3397 can effectively reduce the number of TAMs, inhibit new angiogenesis and ascites production in mesothelioma mouse model, but it does not improve the survival time (Dammeijer et al., 2017). PLX3397 played a good antitumor effect in sarcoma, promoting the proportion of CD8^+^ T, reducing the proportion of FOXP3^+^ CD4^+^ T cells, and increasing the level of CCL2 (Fujiwara et al., 2021).

In lung squamous cell carcinoma, the combination of PLX3397 and PD-1 antibody can increase the contact probability between tumor cells and CD8T cells and delay tumor growth (Peranzoni et al., 2018). PLX3397 monotherapy can promote the CD8T cells tumor infiltration. PLX3397 combined with anti-PD-1 therapy can further increase the accumulation of CD8T in the tumor and delay the progression of the tumor. PLX3397 can inhibit macrophage-mediated T cell exclusion, increase tumor surveillance by CD8 T cells and make PD-1 treatment more effective (Peranzoni et al., 2018). The crosstalk between TAM and T cells is still not fully elucidated in previous studies about the combination of PLX3397 and PD-1 antibody. Therefore, we intend to analyze the underlying mechanism of TAM inhibition to enhancing the efficacy of PD-1 antibody in the process of combination therapy.

## Materials and methods

### Cell line, tumor development and treatment strategy

Lewis lung carcinoma (LLC) was obtained from American Type Culture Collection (ATCC) (Sakai et al., 2006). LLC cell was maintained in DMEM+10% FBS+1% P/S, culture in 5% CO_2_, 37°C. Six to eight-week-old C57BL/6J mice were purchased from GemPharmatech Co., Ltd. The study protocols were approved by the Institutional Animal Care and Use Committee, Sun Yat-Sen University. Mouse was shaved and subsequently inoculated with 10^6^ LLC subcutaneously. After tumor volume reached to 100 mm^3^, the treatment was performed. For anti-CSF1R antibody (BP0213, BioXCell, West Lebanon, NH, United States) or anti-IL-6 antibody (BE0046, BioXCell) neutralization, 50 μg/mouse of each antibody was given through i. v. Injection every 2 days. For PD-1 antibody (BP0146, BioXCell), mice were injected intraperitoneally 200 μg in PBS per mouse every 3 days, *InVivo*Plus rat IgG2a (BP0089, BioXCell) was used as isotype control both for anti-CSF1R antibody group (Isotype: Rat IgG2a, κ) and anti PD1 group (Isotype: Rat IgG2a, κ). For PLX3397 (S7818, Selleck, Shanghai, China), mice were fed with chow containing PLX3397 (50 mg/kg), every 2 days. In the CCL22 compensation experiment, 100 ng/mice of recombinant Mouse CCL22/MDC Protein (439-MD-025, R&D Systems, MN, United States) was given through i. v. Injection at the time of PLX3397 treatment. Mice were sacrificed when the tumor volumes reached approximately 2000 mm^3^ (tumor volume = 1/2 × a × b2; a = length and b = width), and tumor tissues were collected for subsequent experiments.

### Cytometric bead array (CBA) and ELISA

For multiple cytokine analysis, Mouse Th1/Th2/Th17 CBA kit (560485, BD Bioscience, CA, United States) was used as instruction described. Mixed capture beads were added into each test tube, and 50 μl diluted serum sample (serum sample was diluted 1:4) was added into each tube. Then, 50 μl mouse Inflammation PE detection reagent was added all tubes, and incubate for 2 h at room temperature in the dark. 1 ml washing buffer was added to each tube, centrifuge at 200 *g* for 5 min, carefully absorb the supernatant, add 300 μl washing buffer to re-suspend the capture beads. The data collection starts from the low level of standard sample to high, and ensure that the vortex for 3–5 s before sample loading; The BD FACS Canto II flow cytometer was used for data collection.

For mouse chemokine analysis, 50 μl serum was used for ELISA assay after dilute twice using PBS. Mouse MCP-1 ELISA (CCL2) (432704, Biolegend, San Diego, CA, United States), Mouse CCL22/MDC DuoSet ELISA (DY439, R&D Systems, MN, United States), Mouse CXCL9 ELISA Kit (ab203364, Abcom, Atlanta, GA, United States), Mouse IP-10 ELISA Kit (CXCL10) (ab260067, Abcom), were used for chemokine analysis. Optic density was subsequently measured at 450 nm.

### Fluorescence *in situ* hybridization (FISH)

For probe for the Ccl22 (NM_009137.2), the probe sequence and tag design are as follows: 5—FITC—GGACATGCATGGGCAGTGAGCAAAGTAGCA—FITC—3. For the detection, the tissue is obtained and washed using PBS, then immediately placed in the fixation solution (prepared with DEPC water) for 2–12 h. After the tissue fixation, it is dehydrated by gradient alcohol, then immersed in wax and embedded, after section was prepared and incubated in 62°C oven for 2 h. Sections was incubated into xylene for 15 min twice, 100% ethanol for 5 min twice, 85% alcohol for 5 min, 75% alcohol for 5 min, then the section was washed with DEPC. The sections maintained in the repair solution in 100°C for 10–15 min and cooled naturally. Protease K (20 μg/mL) was added for digestion at 37°C for 30 min and then washed with PBS for 3 times. Pre-hybridization solution was added and incubated at 37°C for 1 h The pre-hybridization solution containing probe (8 ng/μl) was added and incubated overnight at 37°C in an incubator. After washed with SSC, sections were stained with DAPI dye solution (2 μg/mL), and the sections were sealed for observation, the data was collected using a fluorescent microscope (DMI8, Leica, Wetzlar, GER). ImageJ was used for integrated optical density analysis.

### Flow cytometry, sorting and macrophage separation

Mechanical dissociation method was used for single cell suspension preparation. Briefly, after tumor dissection, tumor tissues were rinsed in saline to clean off blood and was minced with scissors into 1–2 mm; Then, tissue was put into 70 μm cell strainer (BS-40-XBS, Biosharp, Anhui, China), rubber tip from syringe was used to grinding tissue, single cell suspension was obtained after filtered from the strainer. Single-cell suspensions prepared from tumor or spleen tissues were washed and stained for cell surface phenotyping using the following monoclonal antibodies: CD45-eFluor 450 (48–0451-82, eBioscience), CD11b-APC cy7 (47–0112-82, eBioscience, San Diego, CA, United States), F4/80-PE (12–4801-82, eBioscience), CD115 (CSF1R)-PE-cy7 (25–1152-82 eBioscience), CD8-PE cy7 (25–0081-82, eBioscience), CD4-FITC (11–0042-85, eBioscience), CD25 PerCP-Cyanine5.5 (45–0251-82,eBioscience), FOXP3-APC (17–5773-82, eBioscience), PE anti-mouse CD194 (CCR4) Antibody (131203, Biolegend, San Diego, CA, United States). Single-cell suspensions were stained with antibodies diluted according to the instruction for 30 min, For CD45-eFluor 450 staining, Rat IgG2b kappa Isotype Control (48–4031-82) was used as isotype control, for CD11b-APC cy7, Rat IgG2b kappa Isotype Control (eB149/10H5) (47–4031-82, eBioscience) was used as isotype control, for F4/80-PE and, Rat IgG2a kappa Isotype Control (eBR2a), PE, eBioscience™(12–4321-80, eBioscience) was used as isotype control, for CCR4-PE, PE Armenian Hamster IgG Isotype Ctrl Antibody (400907, Biolegend) was used as Isotype Control; for CD115 (CSF1R)-PE-cy7 and CD8-PE cy7, Rat IgG2a kappa Isotype Control (eBR2a), PE-Cyanine7, eBioscience™(25–4321-82, eBioscience) was used as isotype control, for CD4-FITC, Rat IgG2a kappa Isotype Control (eBR2a)-FITC, eBioscience™(11–4321-80, eBioscience) was used as isotype control, for FOXP3-APC, Rat IgG2a kappa Isotype Control (eBR2a), APC, eBioscience™(17–4321-81, eBioscience) was used as isotype control, for CD25 PerCP-Cyanine5.5, Rat IgG1 kappa Isotype Control (eBRG1), PerCP-Cyanine5.5, eBioscience™(45–4301-80, eBioscience) was used as isotype control. Individual single-color controls were prepared for compensation adjust; samples were washed and resuspended suing PBS. Flow cytometry data was acquisition using CytoFLEX LX ((Beckman Coulter, Brea, CA). Flowjo 10.0 was used for data analysis. For T cell sorting, the CD3^+^CD45^+^ cells were collected using SONY MA900 (Sony, Tokyo, Japan). An example of the gating strategy is given in Supplementary Figure S1.

Anti-F4/80 MicroBeads UltraPure (130–110-443, Miltenyi Biotec, Auburn, CA, United States) was used for tumor-associated macrophage separation according to the instruction. Briefly, the single cell suspension was prepared from tumor tissue using Tumor Dissociation Kit (130–096-730, Miltenyi). 10^7^ total cell was resuspended in 90 μl buffer and 10 μl Anti-F4/80 MicroBeads UltraPure was added, and incubate for 15 min in the dark at 4°C, cells were washed by adding 1 mL washing buffer, and resuspend cells in 500 μl buffer, cells was applied onto the MS column placed in the magnetic field of the MACS separator. MS column was washed with the 3 × 500 μl washing buffer, then the column was removed from the separator and place it on the collection tube. 1 ml of washing buffer was added to the column and immediately flush out the magnetically labeled cells by firmly pushing the plunger into the column.

#### qPCR

Total RNA from separated TAM was extracted using TRIzol reagent following the manufacturer’s protocol. cDNA was synthesized using the PrimeScript RT Reagent Kit (RR037A, TaKaRa, Beijing, China). Quantitative PCR (qPCR) was performed using SYBR Green Master mix (Roche, Basel, Switzerland). The relative expression of genes was determined by the 2^−ΔΔCT^ method and normalized to mActin expression levels. Gene-specific PCR primers are listed below:

*Ccl22*:F: AGG​TCC​CTA​TGG​TGC​CAA​TGT; R: CGG​CAG​GAT​TTT​GAG​GTC​CA.

Arg*1*:F: CTC​CAA​GCC​AAA​GTC​CTT​AGA​G; R: GGA​GCT​GTC​ATT​AGG​GAC​ATC​A.

*Tgfb1*:F: CCA​CCT​GCA​AGA​CCA​TCG​AC; R: CTG​GCG​AGC​CTT​AGT​TTG​GAC.

*Il10*:F: CTT​ACT​GAC​TGG​CAT​GAG​GAT​CA; R: GCA​GCT​CTA​GGA​GCA​TGT​GG

mActin:F: CGT​GAA​AAG​ATG​ACC​CAG​ATC​A; R: CAC​AGC​CTG​GAT​GGC​TAC​GT.

### Statistical analysis

Data were analyzed by Graphpad 9.0 software (GraphPad, Bethesda, MD, United States). Statistical analysis was performed using unpaired two-tailed Student’s t-test for two group comparation, two-way ANOVA was used for more than two group comparation, two-tailed Mantel-Cox test was used for survival curve analysis, *p* values < 0.05 were considered statistically significant. ****p < 0.0001, ***p < 0.001; **p < 0.01; *p < 0.05.

## Results

### Results one PLX3397 switch TAM from M2-like phenotype to M1-like phenotype

**FIGURE 1:**
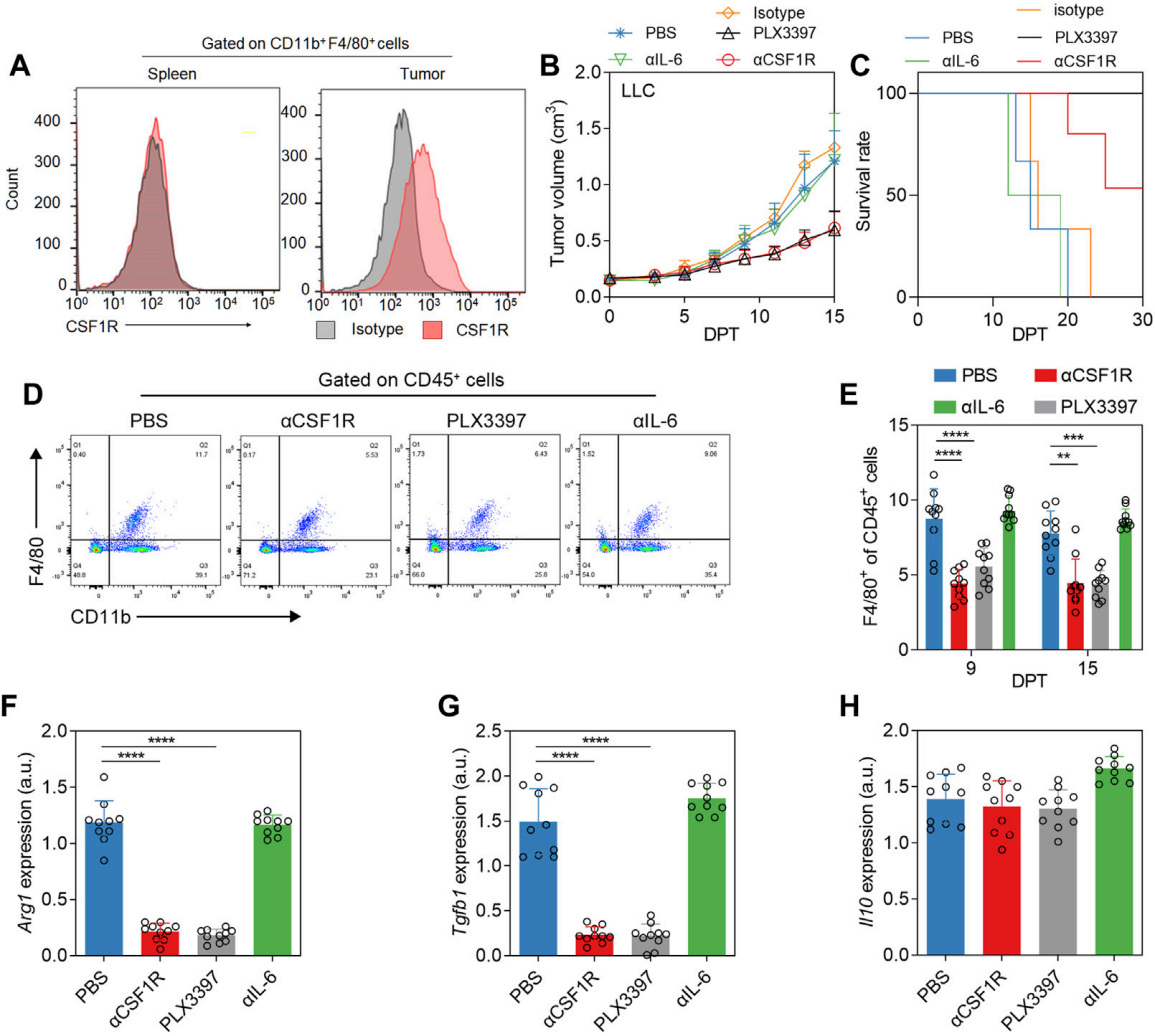
Phenotypic changes of TAM after αCSF1R or PLX3397 treatment. **(A)** Representative flow cytometry histogram of CSF1R levels in macrophages in the spleen or tumor of LLC subcutaneous tumor model, results showed that the CSF1R level in TAM was upregulated. **(B)** When the tumor volume reached 0.1–0.2 cm^3^ on the 7–10 days after subcutaneous tumor inoculation, the treatment experiment was performed according to the description in the method. PLX3397 was administered orally and αCSF1R was administered intravenously. Changes in tumor volume and **(C)** survival of mice were observed, error bar = ±S.D., *n* = 10. **(D)** Representative flow cytometry dot plot of TAM depletion on nine DPT; **(E)** statistical results of TAM depletion on nine DPT and 15 DPT. On 15 DPT, macrophages in the tumor were separated and the transcription levels of **(F)** Arg*1,* **(G)** *Tgfb1* and **(H)** *Il10* were analyzed by qPCR, with error bar = ±S.D.

Firstly, we evaluated the inhibition of TAM using different inhibitors. In addition to PLX3397 (19), a small-molecule inhibitor of CSF1R, CSF1R antibodies have also been validated in a variety of tumor models to inhibit tumor growth by inhibiting the proliferation of TAM(15). We used IL-6 antibody as another candidate for IL-6 also plays an important role in tumorigenesis and progression. It has been reported that macrophage colony-stimulating factors (M-CSF, CSF-1) can significantly promote tumor tissue growth in tumor models constructed from non-small cell lung cancer cell line (Okazaki et al., 2005). We examined the CSF1R phenotype of macrophages in the subcutaneous LLC tumor model. The results showed that TAM had a significantly positive CSF1R phenotype, while macrophages in the spleen of tumor-bearing mice had a negative CSF1R phenotype (Figure 1A). We use αCSF1R (anti-CSF1R antibody), PLX3397, and αIL-6 (anti-IL-6 antibody) to treat subcutaneous lung cancer as method described. The results showed that the αCSF1R neutralization group and PLX3397 treatment group inhibited tumor growth to a certain extent, while there was no significant difference in tumor growth rate between the αIL-6 group and the PBS group (Figure 1B). There was no anti-tumor effect in the αCSF1R isotype control group (Figure 1B). The IL-6 antibody could well neutralize the IL-6 level in the blood (Supplementary Figure S2A), but only played a slight anti-tumor effect, which was consistent with previous reports (Song et al., 2014). In the survival analysis, the survival rate of the α-IL-6 group was similar to that of the PBS group, while the PLX3397 treatment group and the αCSF1R neutralization group reached the humane end point on 27 days post treatment (DPT) began and 25 DPT, respectively (Figure 1C). We analyzed the tumor macrophages in mice on day 7 after treatment began. As expected, the percentage of macrophages was significantly lower in the group receiving CSF1R antibody or PLX3397 (Figures 1D,E). We separated the TAM and analyzed the phenotype changes. As the phenotype of macrophages in tumors tends to be M2c subtype, the main markers include *Tgb1, Il10* (Roszer, 2015). The results showed that the levels of Arg*1* and *Tgfb1* decreased significantly after αCSF1R and PLX3397 treatment (Figures 1F,G), but the level of *Il10* did not decrease significantly (Figure 1H), which indicated that the phenotype of TAM changed from M2-like to M1-like during the treatment.

### Result two The αCSF1R or PLX3397 treatment reprogram the proportion of tumor infiltrated T cells

**FIGURE 2:**
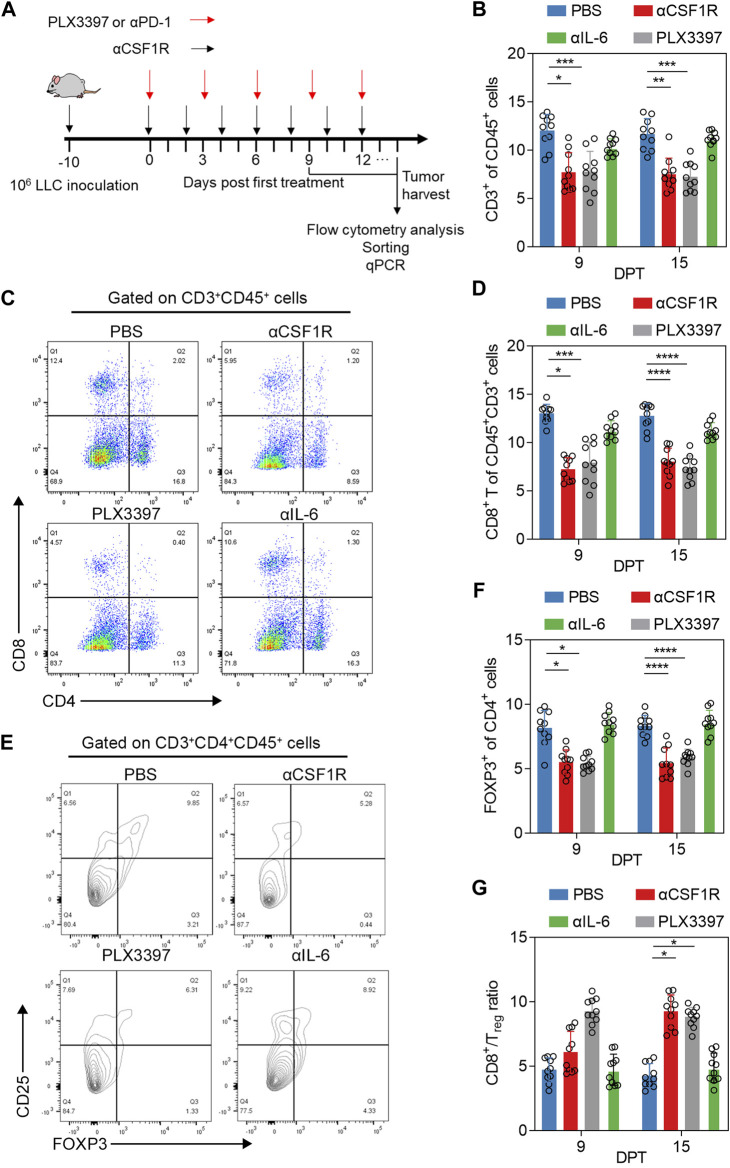
Phenotype and proportion of T cells in tumor after αCSF1R or PLX3397 treatment. **(A)** A graphical representation showed the time line of *in vivo* experiment, 10^6^ LLC cells was inoculated to the right flank of mice, about 10 days post inoculation, the tumor volume would reach about 300 mm^3^ and drug administration begin, the first day of drug administration was recorded as Day 0 post treatment, paratumoral injection was performed as representation described until reached humane endpoint. **(B)** The proportion of CD3^+^ cells in tumor-infiltrating immune cells changed on nine DPT and 15 DPT, the gate strategy was shown in Supplementary Figure S1; **(C)** Representative flow cytometry dot plot about CD8^+^ T of CD3^+^CD45^+^ cells on nine DPT; **(D)** statistical results about CD8^+^ T of CD3^+^CD45^+^ cells on day 9 and 15 after treatment; **(E)** Representative flow cytometry contour blot of Treg cells on day 9 after treatment and **(F)** proportion of Treg cells on 9 and 15 DPT; **(G)** The ratio of CD8^+^ T cells/Treg cells on 9 and 15 DPT.

Due to the important crosstalk between TAM and T cells in tumor immune environment (Bremnes et al., 2016; Cannarile et al., 2017), and in some studies, the use of PLX3397 in nude mice can achieve a better therapeutic effect (Liu et al., 2018; Wu et al., 2020). We examined the changes in the proportion of T cells during PLX3397 and αCSF1R treatment strategy (Figure 2A). The results showed that the ratio of T cells infiltration decreased to a certain extent in the treatment group, and the decrease level in the PLX3397 group was significantly on 15 DPT (Figure 2B). Analysis of CD8^+^ T cells and CD4^+^ cells in tumor after treatment showed that the proportion of CD8^+^ T cells decreased both in 9 and 15 DPT (Figures 2C,D). At the same time, the proportion of Treg cells decreased significantly in both treatment groups on 15 DPT (Figures 2E,F). In the change of immune cells ratio, Treg cells in the αCSF1R group decreased by 72.2% compared with the PBS group (0.18% CD25^+^FOXP3^+^CD4^+^CD45^+^ cells of total CD45^+^ cells in PBS group vs. 0.05% in αCSF1R group) and decreased by 77.8% in the PLX3397 group (0.18% in PBS group vs. 0.04% in PLX3397 group). The ratio of CD8^+^ T cells *versus* Treg cells, a key prognostic factor for cancer, increased in the treated tumors, with a significant increase in levels on both 9 and 15 DPT, with 1.16 times increase in the αCSF1R group compared to the control group (Figure 2G). Next, we want to explore the reasons for the decrease in the proportion of T cells, CD8^+^ T cells and Treg cells during CSF1R targeted therapy. One possibility is that CSF1R targeted therapy acts directly on T cells. The level of CSF1R on the surface of T cells in the tumor was analyzed and shown to be low, so the possibility of CSF1R acting directly on T cells was less likely (Supplementary Figure S2B). The information above indicated that during the inhibition of TAM, the proportion of CD8^+^ T cells and Treg cells has decreased, while the ratio of CD8^+^ T cells *versus* Treg cells was significantly increased. A previous article about CSF1R^+^ macrophages and Foxp3^+^ Treg cells, showed that a slight increase in both CD8^+^ T cells and Treg cells in tumors after PLX3397 treatment (Gyori et al., 2018). One possible reason is that the tumor model and the time point is vary between two research.

### Results three PLX3397 treatment decreased tumor infiltrated treg though reducing TAM-derived CCL22

**FIGURE 3:**
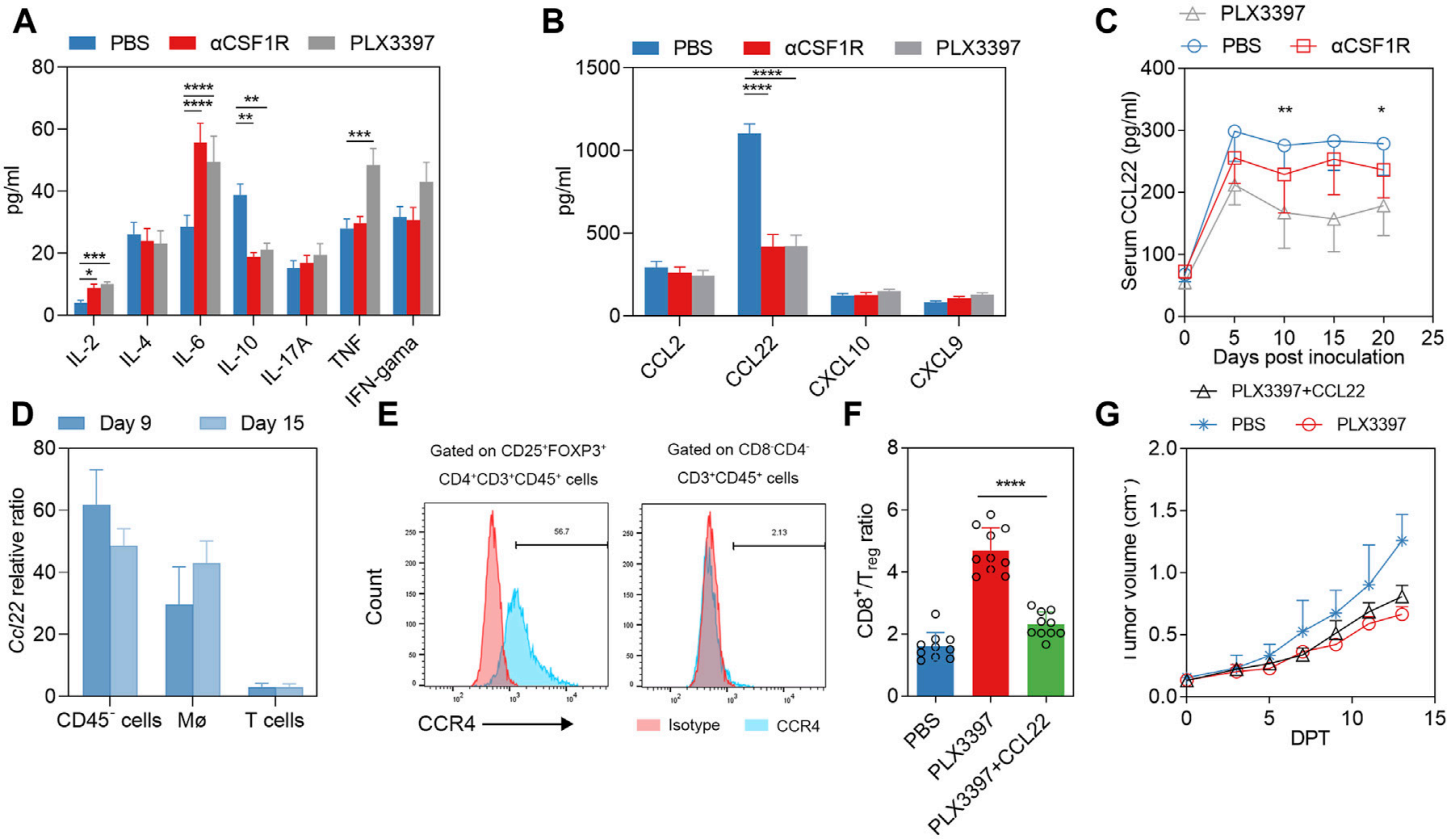
PLX3397 and αCSF1R reduce TAM-derived CCL22 that affect T cell recruitment. **(A)** 15 days after treatment began, serum of mice in each group was collected and the CBA method was used to detect Th1/Th2/Th17-related inflammatory factors, *n* = 10. **(B)** 15 days after treatment began, serum chemokine levels of CCL2, CCL22, CXCL10 and CXCL9 were detected by ELISA kit, *n* = 10. **(C)** Serum levels of CCL22 at day 0,5,10,15 and 20 during the development of murine subcutaneous tumors, with treatment starting at day 7 after tumor inoculation, *n* = 10. **(D)** Macrophages, T cells and CD45^−^ cells within the tumor on 9DPT and 15 DPT were sorted and the overall transcription levels of these cells were analyzed. **(E)** Representative flow cytometry histogram of CCR4 levels in Treg in tumor of LLC subcutaneous tumor model. **(F)** CD8T/Treg ratio at 13 DPT treated with CCL22 combined with PLX3397; And **(G)** tumor volume changes in during the treatment, *n* = 10.

The proportion of T cells in tumor immune cells is also influenced by several other factors. Cytokines and chemokines also have significant influence on T cell infiltration, including CXCL9, CXCL10, CCL20, CCL22, CCL2 and CCL5, *etc.* (Nagarsheth et al., 2017). Treg cells can be induced by a variety of chemokines, such as CCR4‐CCL17/22, CCR8‐CCL1, CCR10‐CCL28 and CXCR3‐CCL9/10/11. Cytokines/chemokines involved in T cells infiltration were detected in the serum on 15 DPT (Figures 3A,B), and the results showed that changes in IL-4, IL-17A and IFNγ were not significant. IL-2 and IL-6 increased after αCSF1R treatment, IL-2 and TNF increased after PLX3397 treatment, and serum IL-10 decreased from 40 pg/mL to 20 pg/mL in both treatment group (Figure 3A). The analysis of chemokines related to T cell chemotaxis showed that CCL2 has no significant change after treatment, CXCL10 and CXCL9 were a little upregulated after PLX3397 treatment (Figure 3B). The level of CCL22 with the highest relative level in PBS group, was significantly decreased in the treatment group (1114.7 pg/mL vs. 493.54 pg/mL vs. 517.16 pg/mL) (Figure 3B). Previous reports have shown that CCL22 is a cytokine from macrophages that induces chemotactic activation of T cells, and its receptor is CCR4, which is mainly expressed on activated CD4^+^ T and Treg cells (Iellem et al., 2001; Sather et al., 2007). It has been reported that tumor-derived CCL22 can recruit Treg cells and promote tumor development. We further identified the time course of serum CCL22 change from the beginning of tumor inoculation. CCL22 has a relatively high level in the early stage of tumor development. After treatment began (7 days post inoculation), the level of serum CCL22 decreased significantly, and the inhibitory effect of PLX3397 on CCL22 was more acute (Figure 3C).

The sources of cytokines and chemokines may be tumor cells, stromal cells and immune cells (Curiel et al., 2004; Liu et al., 2021). We further sorted potential cell subpopulations to identify the source of CCL22. The results showed that the *Ccl22* transcription level of CD45^−^ cells and macrophages increased gradually with the time of tumor progression, while the *Ccl22* mRNA of T cells remained at a low level (Figure 3D). Previous reports have shown that *Ccl22* is a typical marker of the M2-type phenotype of macrophages (Martinez and Gordon, 2014). It has also been reported that macrophage-derived CCL22 can induce infiltration of Treg cells (Gordon and Martinez, 2010; Wang et al., 2019). In order to make it clear that CCL22 was involved in Treg infiltration during treatment, we analysis the CCR4 level in tumor infiltrated Treg, a relatively high CCR4 level was observed in Treg (Figure 3E), we conducted a compensation experiment. The results showed that the CD8T/Treg ratio in the PLX3397 treatment group with CCL22 was significantly inhibited on 13 DPT (Figure 3F). Meanwhile, the antitumor effect of PLX3397 was partially antagonized by the use of CCL22 protein (Figure 3G). The above information indicates that PLX3397 can improve the CD8T/Treg ratio by inhibiting the TAM-derived CCL22 during tumor therapy.

### Results 4 PD-1 antibody combined with PLX3397 brought better outcome

**FIGURE 4:**
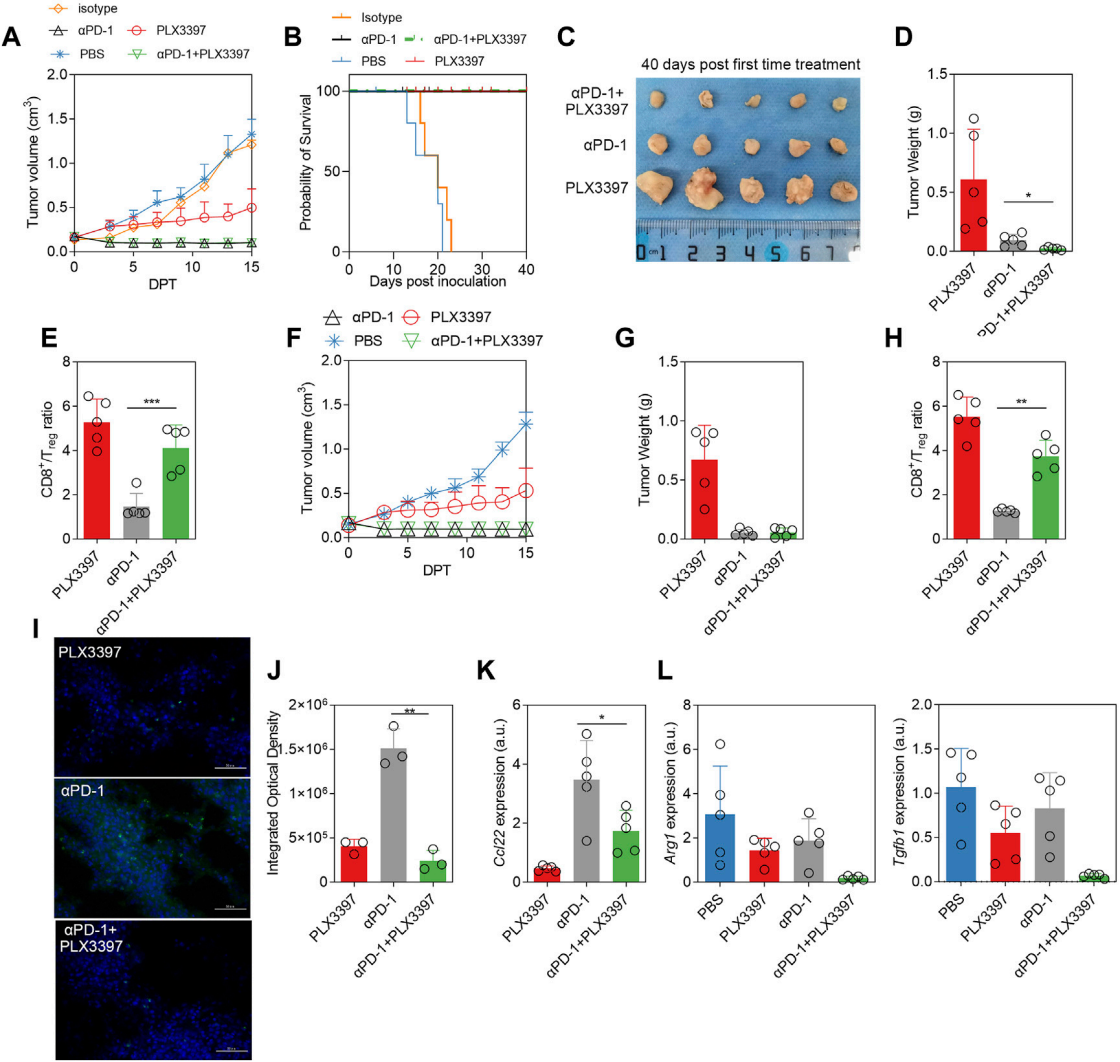
The combination of αPD-1 and PLX3397 showed better therapeutic effect, and αPD-1 further promoted the polarization switch of TAM. **(A)** The change of tumor volume within 15 days after treatment began, *n* = 5. LLC subcutaneous tumor model was developed and the treatment experiment was conducted according to the description in the method; **(B)** Survival rate of different treatment groups after PLX3397 combined with αPD-1, *n* = 5; **(C)** The mice were sacrificed on 40 days after treatment began, the subcutaneous tumors were collected, photographed and **(D)** the tumors were weighed; **(E)** The proportion of CD8^+^ T cells/Treg cells was analyzed by flow cytometry at day 40 after treatment began; **(F)** One of two independent αPD-1 treatment experiments, *n* = 5, **(G)** the tumors of 40 DPT were weighed and **(H)** The proportion of CD8^+^ T cells/Treg cells was analyzed by flow cytometry. **(I)** Representative figure of FISH was used to identify *Ccl22* transcription in tumor and **(J)** integrated optical density statistical results. **(K)** qPCR was used to analyze the transcription of *Ccl22* in purified tumor macrophages on day 40 after treatment. **(L)** The transcriptional levels of Arg*1* and *Tgfb1* in separated TAM on day 15 after treatment began were analyzed by qPCR.

Base on the PLX3397 effect on Treg cells, we treated the tumor model with PD-1 antibody in combination with PLX3397 in the subcutaneous LLC tumor model. It showed a similar tumor growth inhibition between the PD-1 monotherapy group and combination groups within 15 DPT (Figure 4A). After 40 days observation, there was a significant improvement in the survival rate (Figure 4B), and a significant reduction in tumor weight (Figures 4C,D). There was no anti-tumor effect in the αPD-1 isotype control group (Figure 4A). After combination therapy, the ratio of CD8^+^ T cells *versus* Treg cells was significantly increased (Figure 4E). Another independent treatment experiments consisting five mice per group showed a similar result (Figures 4F–H). The *Ccl22* transcription level and expression level of in the tumor were analyzed on the 40 DPT, the transcription level (Figures 4I–K) of *Ccl22* were significantly decreased. It has been reported that PD-1 antibody also has a certain effect on TAM. Our results showed that in the combination treatment group, the polarization level of macrophages was further transformed to M1, and the levels of Arg*1* and *Tgf1b* were decreased significantly on the 15 DPT (Figure 4L).

## Discussion

TAM is an important component of tumor infiltrated immune cells. Various tumor treatment methods are TAM targeted. Researchers used anti-IL-6 antibody (Angevin et al., 2014) and CSF-1R inhibitors (Hollmen et al., 2016) to block the monocytes recruitment into tumor tissue; others by decreasing the population of TAMs, such as anti-CD11b antibody (Zhang et al., 2015). There are also some treatments that use immune activators to transform TAM into M1 proinflammatory macrophage (Le et al., 2012) or attenuate M2 function, such as Trabectedin (Duffaud et al., 2016).

CSF1R is considered to be a good immunotherapeutic target for NSCLC through bioinformatics analysis results (Qi et al., 2020). CSF1R inhibitors have been evaluated in a variety of tumors, including lung squamous cell carcinoma (Cannarile et al., 2017). In this study, CSF1R antibody or PLX3397 treatment could inhibit the macrophages ratio in the tumor, but this treatment could not completely delete TAM. The possible reason is that after drug treatment, the phenotype of macrophages in the tumor showed obvious M1 switch (Figures 1F–H), and CSF1R was more highly expressed in M2-like macrophages. This suggests that CSF1R-targeted inhibitors are more suitable for regulating the tumor immune environment rather than acting as monotherapy. CSF1R inhibitors as monotherapy does not always inhibit tumor growth in some cancer treatment studies, which may be due to individual heterogeneity, immune environment differences and different tumor stages. In our experiment, CSF1R inhibitors as monotherapy showed that a tumor growth inhibition but not tumor volume reduction within 15 DPT (Figure 1B). Therefore, it is necessary to combine CSF1R inhibitor with other immune agents.

In the process of CSF1R treatment, not only TAM is significantly inhibited, other immune cells and immune factors in the tumor immune microenvironment are also significantly changed. Among immune cells, CD8T cells, as terminal antitumor effector cells, and Tregs, which are involved in tumor development, have been shown to be closely associated with TAM (Mosely et al., 2017). Some information shows that TAM can not only promote angiogenesis and support tumor growth by secreting cytokines such as TGF-β (Dirkx et al., 2006), but also recruit T cells by secreting CCL17 and CCL22 (Qian and Pollard, 2010; McAllister and Weinberg, 2014). TAM can also express programmed cell death ligand 1 (PD-L1),CD80, CD86 and other molecules to inhibit the function of CD8T (Mantovani et al., 2017). TAM can even directly inhibit T cell function *in vitro* (Ruffell and Coussens, 2015). It has been reported that small molecule inhibitors can restore the antitumor function of T cells and inhibit tumor growth by eliminating CSF1R^+^ TAM (Candido et al., 2018). Inhibition of the proportion and function of TAM can enhance the cytotoxic effect of cytotoxic T lymphocytes in tumors (DeNardo et al., 2011). In our results, when CSF1R or PLX3397 was used as monotherapy, CD8T cell, CD4T cell and Treg levels in tumor were decreased (Figures 2C–F), which may be caused by the decrease of macrophage-derived chemokine. However, it is worth noting that the level of CD8T/Treg increased significantly (Figure 2G). This phenomenon also indicated that TAM and Treg may have a dynamic balance in tumor immune envrionment.

NSCLC cells and immune cells in TME can inhibit antitumor T cells in a variety of ways, including through coinhibitory receptors such as PD-1. Pharmacological antibodies targeting PD-1 or its ligand PD-L1 can alleviate the inhibition of antitumor T cells, leading to an immune attack on the tumor (Pardoll, 2012) PD-1/PD-L1 therapy could overcome the immunosuppression status of tumor infiltrated T cell. However, how to avoid T cell exhaustion is another bottleneck for T cells in tumor immunotherapy. In our results, PD-1 antibody treatment could not increase the level of CD8/Treg to a relative high level (Figure 4E), and the αPD-1 treatment seems had no effect on macrophage polarization (Figure 4L).

Recent studies have shown that the combination of PD-1 and IL-2 can effectively reverse the exhaustion of CD8T (Hashimoto et al., 2022). In the tumor immune environment, many tumor-infiltrating T cells are naive T cells. How to improve the proportion of tumor specific T cells, reduce the differentiation of T cells into Tregs and the invasion of Tregs into tumors is also worthy of consideration. Woods DM et al. evaluated the predictive significance of Tregs in response to nivolumab in melanoma patients and reported that Tregs showed reduced inhibitory activity in responding patients (Woods et al., 2018). PD-1 therapy not only plays a role in T cells, but also plays a role in macrophages. The level of PD-1 is significantly increased in M2 tumor-infiltrating macrophages in mice. After the use of PD-1 antibody, the phagocytosis ability of these macrophages is significantly improved, thus exerting anti-tumor activity (Gordon et al., 2017).

Pexidartinib was approved by the FDA as monotherapy for symptomatic tenosynovial giant cell tumor (TGCT) associated with severe morbidity or functional limitations and not amenable to improvement with surgery (Lamb, 2019). In Phase I clinical trial, Pexidartinib was combined with sirolimus for the treatment of unresectable Sarcoma and Malignant Peripheral Nerve Tumors (Manji et al., 2021). Pexidartinib was also combined with other anti-tumor drugs like PD-1 checkpoint to approach a better outcome, in Esophageal adenocarcinoma rat model, the use of pexidartinib can improve the efficacy of PD-1 immune checkpoint, Endpoint tissue gene expression results showed that transcription levels of both TGFβ and Arg1 were significantly decreased, which was consistent with our results (Omstead et al., 2022). Novel drug release strategies have also been reported to improve the effect of pexidartinib combined with PD-1 treatment (Li et al., 2022). In these reports, the ratio and activation level of CD8^+^ T cell were further recovered after PD-1 and Pexidartinib combination treatment. In our experiment, the ratio of CD8/Treg was recovered significantly after pexidartinib (PLX3397) combined with αPD-1 (Figures 4E,H), which also indicated that pexidartinib could regulate CD8^+^ T cell function in tumor microenvironment.

CCL22 is a macrophage-derived chemokine expressed in both humans and mice. It is also called macrophage-derived cytokine or stimulated T cell chemotactic protein-1 in human, and B cell-derived chemokine-1 or DC and B cell-derived chemokine in mouse. In addition to macrophages as CCL22’s main source (Korbecki et al., 2020), other sources of CCL22 include activated B cells, mature DC cells, and mouse Langerhans cells (Korbecki et al., 2020). Since tumor cells usually was the largest proportion cell type in tumor tissues, tumor-derived CCL22 cannot be ignored. In this study, the proportion of CCL22 transcription level in tumor cells was similar to that in macrophages (Figure 3D). If the tumor volume shrinks or stagnates during treatment, the level of CCL22 may decrease accordingly. During the CCL22 compensation experiment, the ratio of CD8T/Treg showed a slight increase (Figure 3F). This may indicate that the chemotaxis of CCL22 to Treg is stronger than that of CD8T, which was consistent with previous report, the primary role of CCL22 is to chemotaxis of activated T cells (Korbecki et al., 2020), mainly including Th2 T cells and CD4^+^CD25^+^ regulatory T cells (Iellem et al., 2001). An important article in 2004 has demonstrated that neutralizing CCL22 can inhibit Treg infiltration and further inhibit tumor growth (Curiel et al., 2004). Researchers reported that CCL22 can recruit CD8^+^T lymphocytes in the tumor microenvironment and inhibit tumor growth (Bremnes et al., 2016). However, there is also some literature showing that CCL22 can promote tumor growth. In the tumor microenvironment, CCL22 induces Treg infiltration, which further inhibits the function of CD8T cells and NK cells, forming an immunosuppressive environment and ultimately leading to tumor growth (Korbecki et al., 2020). At the same time, CCL22 can further stimulate Treg to secrete IL-10, which can polarize macrophages to M2 type and promote tumor growth (Chen et al., 2021). In our results, there was a significant decrease in serum IL-10 levels after the use of αCSF1R or PLX3397 (Figure 3A), which may be due to a decrease in the proportion of M2-type macrophages or a decrease in Treg levels. Recent studies have shown that CCL22 can recruit trophoblast cells to support B-cell maturation (Liu et al., 2021); CCL22 may also potentially have a nursing-like effect in the tumor development. The functional duality of CCL22 and the downstream effects on cells and related functions remain unclear. In our study, CCL22 level was significantly inhibited when treated with PLX3397, which further inhibited Treg infiltration in the tumor environment, which improve the anti-tumor effect of PD-1 antibody therapy methods. During the combination treatment, tumor immune environment was reprogramed significantly, TAM polarization is further switched and CD8T/Treg ratio was increased. Our study showed that combination of CSF1R and PD-1 therapy, could work synergistically base on the crosstalk between TAM and CD8T.

## Data Availability

The original contributions presented in the study are included in the article/Supplementary Material, further inquiries can be directed to the corresponding authors.
