# Supplementary material for: Pexidartinib synergize PD-1 antibody through inhibiting treg infiltration by reducing TAM-derived CCL22 in lung adenocarcinoma
Source: Front Pharmacol. 2023 Mar 8;14:1092767. doi: 10.3389/fphar.2023.1092767 (PMC10030616; doi:10.3389/fphar.2023.1092767)
Supplement: Supplementary file 1 [file DataSheet1.DOCX]

Supplementary Material


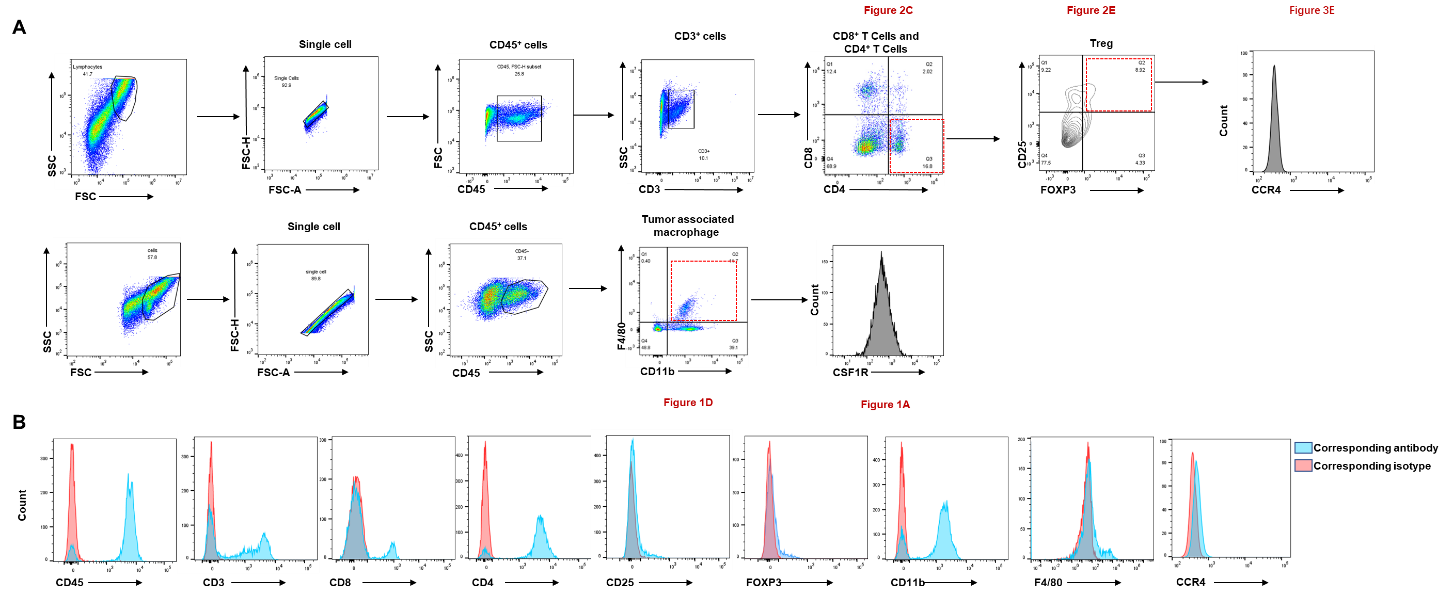


**Supplementary Figure 1.** **(A)** Exemplifying gating strategies for FACS analysis are shown. Gating strategy for tumor-infiltrating CD3^+^CD8^+^ T cells (for Figure 2C) and Treg (for Figure 2E); gating strategy for tumor-associated macrophages (for Figure 1D), CSF1R phenotype analysis (for Figure 1A), CCR4 phenotype analysis (for Figure 3E); **(B)** Representative flow cytometry histograms of each marker with corresponding isotype.


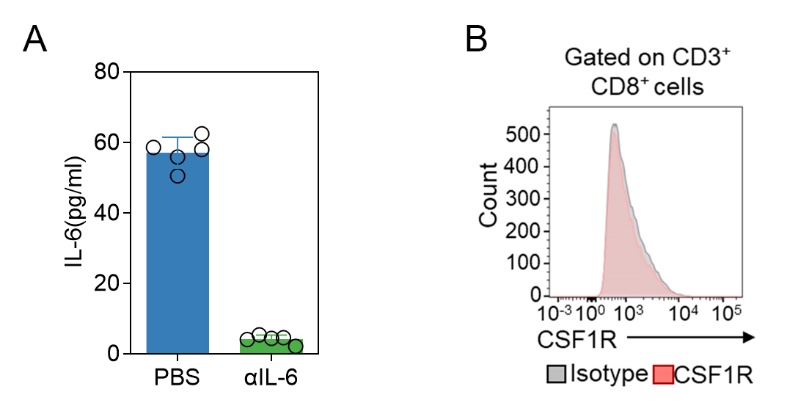


**Supplementary Figure 2.** **(A)** Peripheral blood IL-6 levels after 9 DPT during tumor therapy with αIL-6 antibody; **(B)** Tumor-infiltrating T cell CSF1R phenotype in LLC subcutaneous tumor model;
